# Preoperative Risk Factors and Phenotypic Clustering in Periprosthetic Joint Infection: A Matched Case–Control Study

**DOI:** 10.3390/life15111762

**Published:** 2025-11-17

**Authors:** Tarek Omar Pacha, Sophia K. Klett, Gabi von Lewinski, Maximilian Koblenzer, Hür Özbek, Jorge Mayor, Jan D. Clausen, Axel Gänsslen, Schayan Faraj Tabrizi, Stephan Sehmisch, Gökmen F. Aktas

**Affiliations:** Department of Trauma and Orthopaedic Surgery, Hannover Medical School (Medizinische Hochschule Hannover), Carl-Neuberg-Straße 1, 30625 Hannover, Lower Saxony, Germany

**Keywords:** periprosthetic joint infection (PJI), total joint arthroplasty, preoperative risk factors, phenotypic clustering, machine learning in orthopedics, risk stratification

## Abstract

Periprosthetic joint infection (PJI) remains one of the most serious complications after total joint arthroplasty. This retrospective 1:1 matched case–control study investigated preoperative predictors and patient phenotypes associated with PJI in 182 patients (91 cases, 91 controls) undergoing hip or knee arthroplasty between 2013 and 2024. Variables with skewed distributions were log-transformed, and multivariable logistic regression with LASSO regularization identified independent risk factors. Unsupervised K-means clustering was applied to perioperative features to explore data-driven patient phenotypes. Preoperative anemia (OR 5.91, *p* = 0.026), higher ASA score (OR 1.77, *p* = 0.033), and surgical delay (OR 1.67, *p* = 0.024) independently predicted infection, while age and CRP showed non-significant trends. The resulting five-variable preoperative model achieved an AUC of 0.718 (optimism-corrected AUC of 0.661) for infection prediction. Clustering analysis revealed three phenotypes: anemia-dominated, elderly but short-procedure, and high surgery duration with significantly different infection rates (χ^2^ = 23.5, *p* < 0.001) but similar mortality (*p* = 0.068). This integrative approach combining regression-based prediction and phenotype discovery enables clinically interpretable, preoperatively applicable risk stratification. The findings identify anemia, comorbidity burden, and surgical delay as key modifiable targets for preventive optimization before arthroplasty. External validation and recalibration to population-level incidence are warranted before clinical implementation.

## 1. Introduction

Periprosthetic joint infection (PJI) is still rising [1] and remains a devastating and serious complication after total joint arthroplasty [2,3], leading to repeated revision surgeries [4], prolonged hospital stays, impaired functional outcomes, and increased mortality [5]. The economic burden of such complications is substantial and was estimated with an amount of USD 566 million in 2009 in the US [4]. Despite advances in surgical techniques, perioperative antibiotic prophylaxis, and enhanced recovery pathways, infection continues to place a substantial burden on patients and healthcare systems [6]. Due to the increasing complexity of patients, PJIs impact more than 2% of the patients receiving arthroplasty [1,7,8]. Current evidence hints at some well-established predictors, like prolonged operative duration, higher ASA scores, and anemia [9]. However, the impact of additional perioperative factors, including preoperative anemia, elevated inflammatory markers, and surgical delay, to our knowledge, remains less well defined. Importantly, robust multivariable prediction models that allow individualized patient risk assessment are still lacking in routine clinical practice. In recent years, complex machine learning models incorporating 60 or more variables have been proposed, and these approaches consistently converge on a core set of predictors such as operative duration, ASA-Score, and anemia [9,10]. While these models demonstrate strong performance, their high dimensionality limits feasibility for daily clinical use [9]. The present study, therefore, seeks to apply regression analysis, aiming to achieve comparable predictive utility with a substantially reduced set of features. The goal is to provide a patient-specific feature set that can be rapidly obtained and easily applied in daily clinical practice, while preserving the data-driven foundation. Beyond infection risk alone, our approach explicitly integrates mortality prediction through receiver operating characteristic (ROC) analysis and feature-guided clustering techniques to identify distinct patient phenotypes based on perioperative characteristics.

## 2. Methods

### 2.1. Study Design and Population

We conducted a retrospective 1:1 matched case–control study at a single tertiary university hospital. From all patients who underwent hip or knee arthroplasty between 2013 and 2024, we identified 97 patients who developed a confirmed primary periprosthetic joint infection (PJI cases). Controls were selected using 1:1 greedy matching based on age (±5 years) and exact sex match. The final matched cohort comprised 91 PJI cases and 91 infection-free controls, resulting in a study population of 182 patients (case-to-control ratio 1:1). Statistical testing confirmed successful age balance (Mann–Whitney U test, *p* = 0.92) and perfect sex balance (Chi-square test, *p* = 1.00) between groups. This case–control design resulted in a study population with 50.0% infection rate by design, which substantially exceeds the true epidemiological incidence of PJI (1–3% in routine clinical practice) [7]. Our findings, therefore, reflect risk factor associations within a 1:1 matched case–control framework rather than population-based infection rates. Importantly, only primary postoperative infections arising within our own institutional cohort were included, while patients with secondary infections or referrals from external hospitals were excluded.

A strict post hoc classification according to the 2018 ICM/MSIS criteria was not feasible due to the retrospective nature of data collection. Several diagnostic parameters required for minor-criteria scoring, such as α-defensin testing, synovial CRP, and D-dimer, were not routinely obtained in all cases during the study period. Consequently, a full recalculation of the ICM/MSIS score for each patient would have introduced substantial selection bias and data loss.

For this reason, the present analysis applied a pre-specified clinical definition consistent with the institutional diagnostic routine at that time, which was designed to maximize real-world sensitivity while maintaining clinical validity.

Therefore, periprosthetic joint infection was defined as any infection with intraoperatively confirmed microbial growth from periprosthetic samples or clinically evident infection confirmed during surgery. In contrast to the 2018 ICM/MSIS criteria, which require a sinus tract, at least two concordant positive cultures, or additional confirmatory findings as major criteria, our definition is more sensitive but less specific. While age and sex were successfully balanced through matching, significant differences remained for several perioperative variables, including operative duration, baseline CRP, ASA score, surgical delay, anemia, and mortality (Mann–Whitney U or Chi-square tests, all *p* < 0.05). These variables, together with postoperative antibiotic prophylaxis and surgical localization (hip vs. knee arthroplasty), were included as candidate predictors in the regression model. In addition to regression-based risk prediction, we performed feature-guided clustering analysis to identify distinct patient phenotypes based on perioperative characteristics. Clustering was performed independent of outcome status to avoid circularity, using the same perioperative predictors to ensure consistency between analytical approaches.

### 2.2. Variable Coding and Transformations

Continuous variables with skewed distributions (operative time, baseline CRP, surgical delay, age) were natural log-transformed (ln) to approximate normality for clustering analysis, as confirmed by Shapiro–Wilk testing (all *p* < 0.05, Table 1). Binary covariates were coded as 1 = present and 0 = absent. Anemia was defined as preoperative hemoglobin concentration < 10 g/dL [11,12]. Postoperative antibiotic prophylaxis was defined as any intravenous antibiotic administration given after completion of surgery. Surgical localization (hip vs. knee arthroplasty) was coded as a binary variable (knee = 1, hip = 0). ASA physical status score was treated as an ordinal variable (range 1–4). Perioperative mortality was defined as death during the index admission or subsequent treatment-related readmissions (yes = 1, no = 0). For regression analysis, odds ratios for binary predictors represent the change from absence (0) to presence (1).

**Table 1:** Descriptive statistics of key study variables. Descriptive statistics for continuous variables in the study cohort. Data are presented as minimum, first quartile (Q1), median, third quartile (Q3), maximum, mean, and standard deviation. All variables except age demonstrated significant skewness, necessitating log-transformation for statistical analysis.

Variable	N	Miss	Mean	SD	Median	Q25	Q75	Min	Max
Age (years)	182	0	72.01	11.40	72.00	65.00	80.75	43.00	95.00
Surgery duration (min)	182	0	130.80	75.52	109.00	82.25	144.75	51.00	501.00
CRP (mg/L)	182	0	18.15	30.29	5.00	2.30	17.40	0.00	168.00
Delay of surgery (days)	182	0	2.12	5.42	1.00	0.00	1.00	0.00	54.00
Duration in hospital (days)	182	0	14.16	13.29	10.00	7.00	15.00	2.00	98.00
ASA score	182	0	2.57	0.64	3.00	2.00	3.00	1.00	4.00

### 2.3. Acknowledgement of AI Assistance

The authors acknowledge the use of artificial intelligence (AI) tools (ChatGPT 5, OpenAI, San Francisco, CA, USA) for editorial assistance. AI support was limited to language-related services, including grammar correction, translation, and improvements in style and structure. No AI system was involved in generating scientific content, data analysis, interpretation of results, or drawing conclusions. All intellectual responsibility for the content of this manuscript rests solely with the authors.

### 2.4. Ethics Statement

This study was conducted in accordance with the Declaration of Helsinki and approved by the institutional ethics committee (reference number 11181_BO_K_2023). Owing to the retrospective design, the requirement for individual informed consent was waived.

### 2.5. Statistical Analyses

Normality Testing: Shapiro–Wilk tests were performed on continuous variables to assess appropriateness for K-means clustering analysis, which assumes approximately normal distributions for optimal performance. Five of six core variables demonstrated significant deviations from normality (all *p* < 0.05, Table 2), justifying log-transformation. Note that normality testing is not required for logistic regression, which makes no assumptions about predictor distributions; thus, we considered normal distribution, being ideal for cluster analysis.

**Table 2:** Normality assessment of continuous variables using Shapiro–Wilk tests. This table presents the results of normality testing for all continuous variables included in the analysis. All variables demonstrated significant deviations from normality (*p* < 0.05), justifying the use of log-transformations and non-parametric statistical methods throughout the study.

Variable	Shapiro-W	*p*-Value	Normality
Age (years)	0.9671	0.0003	no
Surgery duration (min)	0.9582	0.0000	no
CRP (mg/L)	0.9739	0.0017	no
Delay of surgery (days)	0.8218	0.0000	no
Duration in hospital (days)	0.6370	0.0000	no

Group Comparisons: Mann–Whitney U tests were used for continuous variables and Chi-square (or Fisher’s exact) tests for categorical variables when comparing baseline characteristics between cases and controls.

Regression Analysis: Multivariable logistic regression was used to identify independent predictors of periprosthetic joint infection. Ridge and LASSO regularization were performed separately to address multicollinearity. The final full model included seven variables selected through LASSO regularization from nine candidate predictors. Model performance was assessed using receiver operating characteristic (ROC) curves with area under the curve (AUC) as the primary metric.

Discrimination was quantified by the area under the receiver operating characteristic curve (AUC). Given the 1:1 matched case–control design, prevalence-dependent metrics such as PPV or NPV were not reported. Calibration was assessed using calibration plots, the Brier score, and the Hosmer–Lemeshow goodness-of-fit test. Clinical utility was evaluated using decision curve analysis (DCA). To reflect real-world conditions, net benefit was calculated with prevalence weighting using a target infection prevalence of 2% (with 1–3% incidence reported in the literature). Internal validation used bootstrap resampling (1000 iterations) with model refitting in each iteration to estimate optimism.

The optimism-corrected AUC was computed as the apparent AUC minus average optimism.

Cut-off Analysis: The optimal probability thresholds were determined using Youden’s index, identifying the threshold maximizing the overall classification accuracy. We acknowledge that this approach assumes equal costs for false-positive and false-negative predictions and does not account for disease prevalence. Sensitivity and specificity) were calculated for different thresholds to facilitate clinical interpretation across different risk tolerance scenarios.

Clustering Analysis: Unsupervised K-means clustering was performed on z-score standardized perioperative features to identify natural patient phenotypes independent of infection status. The optimal number of clusters was determined using silhouette analysis, with k = 3 achieving the highest silhouette score (0.308). To avoid circularity, cluster validation focused exclusively on outcome variables (infection status, mortality). Chi-square tests evaluated whether clusters demonstrated statistically significant differences in clinical outcomes.

Dimensionality Reduction: t-distributed Stochastic Neighbor Embedding (t-SNE) was applied to visualize the clusters in two-dimensional space. This confirms spatial separation between identified phenotypes. All analyses were conducted in Python 3.10 using pandas (data manipulation), numpy (numerical operations), scikit-learn (clustering, outlier detection), statsmodels (logistic regression), scipy (statistical testing), matplotlib, and seaborn (visualization).

## 3. Results

### 3.1. Descriptive Statistics

Cohort Characteristics: The 1:1 matched study cohort comprised 182 patients, including 91 patients with periprosthetic joint infection and 91 infection-free controls. The cohort had a mean age of 72.0 ± 11.4 years (range 43–95), with successful age balance between cases and controls (*p* = 0.92). Mean operative duration was 130.8 ± 75.5 min, baseline CRP was 18.2 ± 30.3 mg/L (median 5.0 mg/L), and mean surgical delay was 2.1 ± 5.4 days (median 1 day). Mean hospital length of stay was 14.2 ± 13.3 days. Overall perioperative mortality was 5.5% (n = 10). The cohort included 96 male (52.7%) and 86 female (47.3%) patients, with 71.4% undergoing hip arthroplasty and 28.6% knee arthroplasty. ASA physical status scores were predominantly ASA 2 and 3, consistent with the tertiary care hospital setting and major joint arthroplasty indication. Preoperative anemia (hemoglobin < 10 g/dL) was present in 23 patients (12.6%), and postoperative antibiotic prophylaxis was administered to 114 patients (62.6%) (Table 1).

### 3.2. Patient Phenotype Identification

**Figure 1:**
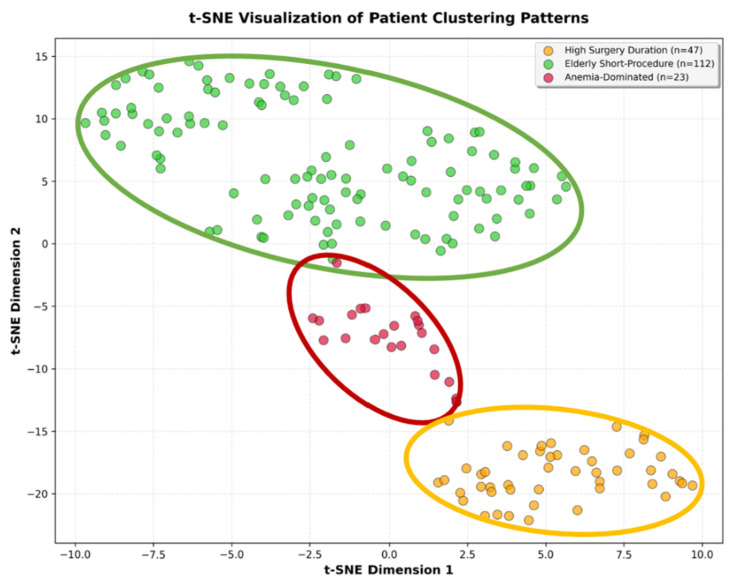
t-SNE visualization of patient clustering patterns. Two-dimensional t-SNE plot visualizing the natural separation of patients based on perioperative characteristics. Each point represents one patient, color-coded by cluster assignment: red for Anemia-Dominated Cases (n = 23), orange for High Surgery Duration (n = 47), and green for Elderly Short-Procedure (n = 112). The clear spatial separation between clusters validates the distinct phenotypic groupings identified through K-means clustering analysis.

Feature-guided clustering analysis using the K-means algorithm identified three distinct patient phenotypes with good cluster separation (silhouette score 0.308). Clustering was performed using perioperative characteristics (age, operative duration, anemia, CRP, ASA score, and surgical delay) independent of infection status to identify natural patient subgroups. Visual inspection using t-distributed stochastic neighbor embedding (t-SNE) confirmed clear spatial separation between clusters (Figure 1).

**Figure 2:**
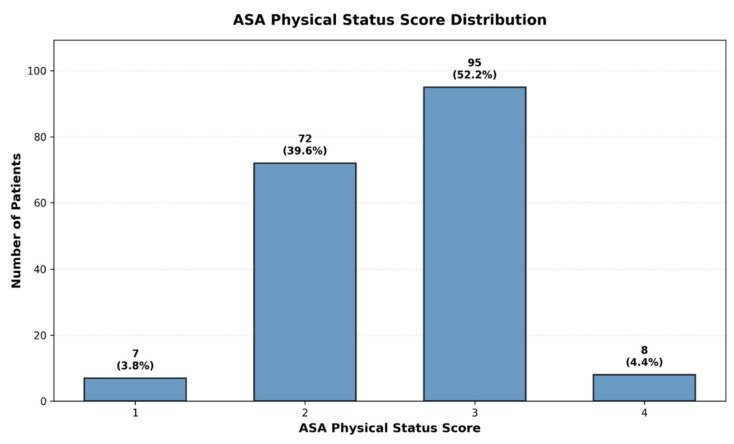
ASA physical status score distribution in the study cohort. Bar chart illustrating the distribution of ASA physical status scores among study participants. The majority of patients had ASA scores of 2 or 3, reflecting moderate to severe systemic disease typical of a tertiary care hospital population.

To validate clinical relevance, we tested whether the identified phenotypes differed significantly in clinical outcomes that were not used for cluster formation. Chi-square analysis showed that the three clusters exhibited statistically significant differences in infection rates (χ^2^ = 23.52, *p* < 0.001) but not in perioperative mortality (χ^2^ = 5.38, *p* = 0.068), confirming that the phenotype classification captures clinically meaningful infection risk stratification (Table 3). Additionally, the majority of patients we included in this study had ASA scores of 2 or 3 (Figure 2).

### 3.3. Cluster 0—“High Surgery Duration” (n = 47)

This intermediate-risk phenotype was distinguished by younger patient age (63 ± 10 years), extended operative times (159 ± 95 min), minimal anemia prevalence (2.1%), and moderate comorbidity burden (mean ASA score 2.4 ± 0.6). Within the matched case–control cohort, zero perioperative mortality was observed despite prolonged surgical procedures, suggesting that younger age and the absence of anemia confer resilience against perioperative mortality.

### 3.4. Cluster 1—“Elderly Short-Procedure” (n = 112)

This largest phenotype comprised the oldest patients (76 ± 10 years) undergoing brief procedures (110 ± 54 min) with no anemia and moderate comorbidity burden (ASA score 3). Within the matched cohort, this cohort showed notable perioperative mortality (6.3%), likely reflecting age-related frailty as an independent mortality risk factor beyond infection status.

### 3.5. Cluster 2—“Anemia-Dominated Cases” (n = 23)

This highest-risk phenotype was characterized by near-universal anemia prevalence (95.7%), elevated comorbidity burden (ASA score 3), mean age 72 ± 10 years, and prolonged operative duration (176 ± 84 min). Within the matched case–control cohort, this cluster exhibited the highest perioperative mortality (13.0%).

Within the matched case–control cohort, anemia-dominated cases (Cluster 2) exhibited the highest risk infection rates and 13.0% perioperative mortality. High surgery duration patients (Cluster 0) showed intermediate infection rates but remarkably achieved zero perioperative mortality despite prolonged procedures, suggesting the protective effects of younger age and absence of anemia. Elderly short-procedure patients (Cluster 1) demonstrated lower infection rates, but notable perioperative mortality (6.3%), highlighting age-related frailty as an independent mortality driver beyond infection status (Table 3).

### 3.6. Inferential Statistics

Multivariable logistic regression using preoperative variables identified three statistically significant independent predictors of periprosthetic joint infection (Table 4). Preoperative anemia (hemoglobin < 10 g/dL) demonstrated the strongest association with infection (OR 5.91, 95% CI 1.23–28.37, *p* = 0.026). Higher ASA physical status scores increased infection odds by 77% per one-unit increase (OR 1.77, 95% CI 1.05–2.99, *p* = 0.033). Surgical delay showed a significant association with infection risk (OR 1.67 per log-unit increase, 95% CI 1.07–2.62, *p* = 0.024). Age and baseline CRP levels showed non-significant trends but were retained in the final model for clinical completeness and to control for potential confounding (Table 4). The final reduced model was specifically designed for preoperative application, excluding operative duration (not known before surgery) and postoperative antibiotic prophylaxis (confounded by indication), to enable risk stratification and optimization before the surgical procedure. The odds ratios derived from this case–control design represent valid estimates of relative risk associations, though absolute risk predictions require recalibration to population-level infection prevalence rather than the prevalence in our matched cohort.

### 3.7. Predictive Model Performance

**Figure 3:**
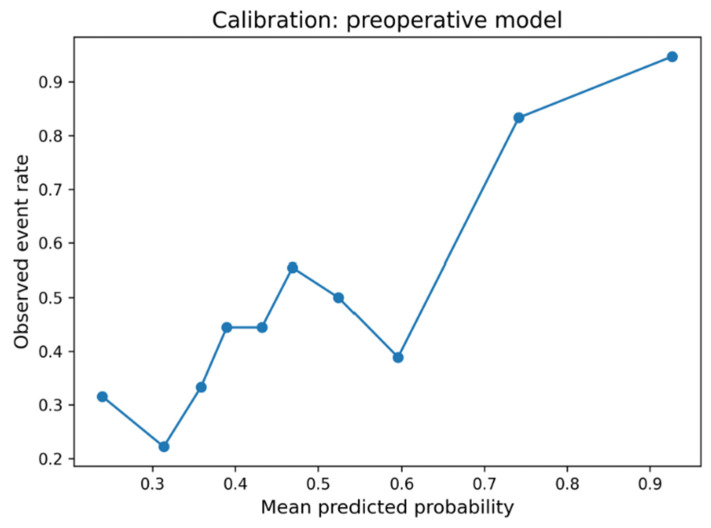
Calibration curve of the preoperative model. Calibration plot of the preoperative model. The observed infection rate closely followed predicted probabilities (Hosmer–Lemeshow χ^2^ = 5.50, *p* = 0.70).

**Figure 4:**
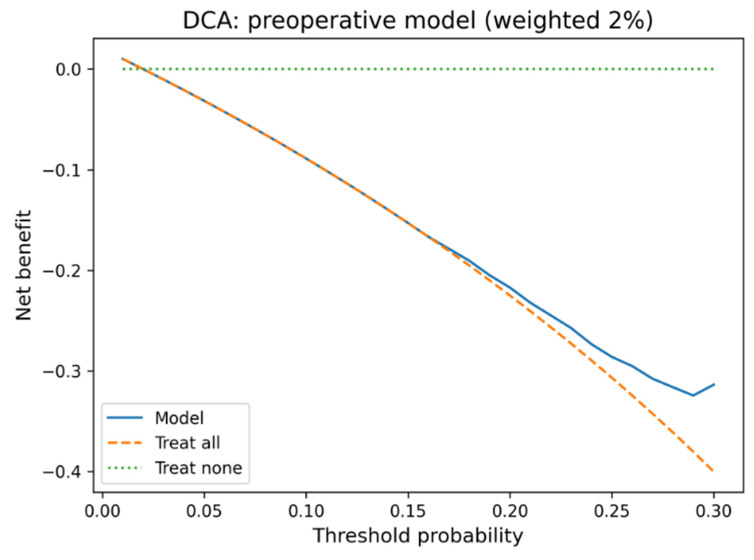
Decision-curve analysis for the preoperative model, prevalence-weighted at 2%. Decision-curve analysis of the preoperative model (prevalence-weighted to 2%). The model provided small positive net benefit compared with “treat all” and “treat none” strategies across threshold probabilities below 0.10.

**Figure 5:**
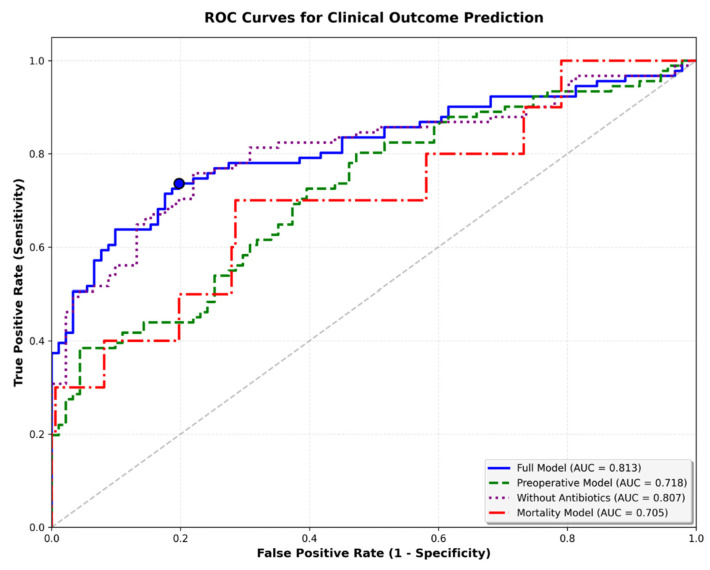
Receiver operating characteristic curves for clinical outcome prediction. ROC curves demonstrating discriminatory performance for two predictive models: preoperative infection prediction (AUC = 0.718) and perioperative mortality prediction (AUC = 0.705). Optimal thresholds according to Youden’s index are marked for each model. Full model with all parameters is shwon as well ( AUC = 0.813).

The five-variable preoperative logistic regression model (preoperative model) achieved moderate discriminatory performance for infection prediction (AUC = 0.718). In contrast, the full model (AUC = 0.813) and the simple reduced model (AUC = 0.807/full model without postoperative antibiotics) perform slightly better than the final preoperative model. This final model was specifically designed to use only information available before surgery, excluding operative duration and postoperative antibiotic prophylaxis. The preoperative model demonstrated acceptable calibration (Brier score = 0.210; Hosmer–Lemeshow χ^2^ = 5.50, *p* = 0.70), whereas the full model achieved slightly better calibration (Brier = 0.172; HL χ^2^ = 9.99, *p* = 0.27). Decision-curve analysis weighted to a real-world infection prevalence of 2% (1–3% Incidence) showed limited but consistent net benefit over “treat all” and “treat none” strategies at low-risk thresholds (<0.10). In internal bootstrap validation (1000 iterations), the preoperative model achieved an apparent AUC of 0.718 and an optimism-corrected AUC of 0.661, indicating moderate discrimination and stable calibration within the limits of the matched case–control design (Figure 3 and Figure 4). The preoperative focus ensures the model can guide preventive strategies and patient counseling before the surgical procedure. Optimal probability threshold according to Youden’s index was 49.6%, achieving balanced sensitivity (73.6%) and specificity (80.2%). However, we acknowledge that Youden’s index assumes equal misclassification costs and does not account for disease prevalence (Table 5, Figure 5). Perioperative mortality prediction (mortality model) using the same preoperative variables yielded an AUC of 0.705, reflecting the relatively small number of events (n = 10 deaths among 182 patients) but still demonstrating clinically meaningful discrimination above chance performance. The lower AUC compared to infection prediction likely reflects both the limited statistical power from few events and the multifactorial nature of perioperative mortality beyond the captured preoperative variables.

### 3.8. Predictive Formula

To enable individualized preoperative infection risk estimation, we deployed the five variables used in the preoperative model to a multivariable logistic regression model incorporating age (log-transformed), anemia, CRP (log-transformed), ASA score, and surgical delay (log-transformed). The model produces a linear predictor (logit score) by combining regression coefficients with patient-specific values, as outlined in Equation (1).

General Logistic Regression:

(1) $$z\;=\;\beta_{0}\;+\;\beta_{1}\times X_{1}\;+\;\beta_{2}\times X_{2}\;+\;...\;+\;\beta_{n}\times X_{n}$$

The resulting preoperative regression equation was:

z = 1.71 − 0.87 × ln(age) + 1.78 × Anemia + 0.12 × ln(CRP) + 0.57 × ASA score + 0.51 × ln(Delay)

The linear predictor (z) represents the logit score, which can be transformed to probability using the logistic function. With Equation (2), the ranked probability for infection can be assessed:

(2) $$P(Infection)\;=\frac{1}{1\;+\;\hat{e}(-z)}\;$$

However, due to the matched case–control design, the logit score (z) provides valid relative risk stratification.

### 3.9. Calculation Example for Preoperative Risk Stratification

A 75-year-old patient scheduled for arthroplasty with preoperative anemia (hemoglobin < 10 g/dL), baseline CRP of 8 mg/L, ASA score of 3, and 1.5-day preoperative delay would have:

Age = ln(75) = 4.32, CRP = ln(8) = 2.08, Delay = ln(1.5) = 0.41, Anemia = 1, ASA_score = 3

Substituting into the equation:

z = 1.71 − 0.87 × 4.32 + 1.78 × 1 + 0.12 × 2.08 + 0.57 × 3 + 0.51 × 0.41 = 1.90

Risk Stratification Using Logit Score:

Rather than converting to absolute probability, we use the logit score for relative risk stratification:

- Low Risk: z < −1.10
- Intermediate Risk: −1.10 ≤ z < 0.00
- High Risk: z ≥ 0.00

This patient’s logit score of z = 1.90 places them in the high-risk category. This preoperative risk assessment enables targeted interventions before surgery, such as systematic anemia correction, comorbidity optimization, and minimization of surgical delay.

## 4. Discussion

This comprehensive analysis of 182 matched patients undergoing total joint arthroplasty reveals that perioperative infection risk is driven by complex interactions between procedure-related and patient-related factors, with distinct patient phenotypes exhibiting markedly different risk profiles. Our dual approach, combining predictive modeling with feature-guided clustering, provides novel insights into both individualized risk estimation and population-based risk stratification.

Preoperative anemia emerged as the strongest modifiable predictor (OR 5.91), emphasizing the critical importance of preoperative hemoglobin optimization [13,14]. Higher ASA scores increased infection odds by 77% per unit increase (OR 1.77), consistent with the established literature demonstrating the impact of comorbidity burden [15]. Surgical delay showed a significant association with infection risk (OR 1.67), highlighting the importance of expedited surgical scheduling for high-risk patients.

Our model was specifically designed for preoperative application. While previous studies have identified operative duration as one of the strongest risk factors for PJI [16,17], this variable was not available preoperatively and therefore could not inform preoperative optimization strategies or patient counseling. Our analysis confirmed this finding in retrospective analysis (OR 7.54 when included), but, in the end, we excluded it from the clinical prediction model to focus on modifiable preoperative risk factors. Furthermore, current evidence does not support prolonged postoperative antibiotic prophylaxis beyond 24 h [7,18,19], and most likely, any observed association reflects patient selection rather than a causal effect; thus, the postoperatively given antibiotic prophylaxis was excluded as well. In consequence, the preoperative focus of our model enables identification of high-risk patients before surgery, allowing targeted interventions, such as systematic anemia correction, comorbidity stabilization, and delay minimization. This represents a clinically actionable approach to infection prevention, focusing exclusively on factors that can be optimized before surgery.

Feature-guided clustering analysis revealed three distinct patient archetypes with divergent clinical trajectories. Within the matched case–control cohort, anemia-dominated cases (12.6% of cohort) represented the highest-risk phenotype, characterized by near-universal anemia, prolonged procedures, and extremely high infection rates with 13.0% perioperative mortality. High surgery duration patients (25.8% of cohort) demonstrated intermediate infection risk but remarkable resilience with zero perioperative mortality despite procedural complexity, suggesting protective effects of younger age and absence of anemia. Elderly short-procedure patients (61.5% of cohort) exhibited notable age-related perioperative mortality (6.3%), highlighting age as an independent mortality driver beyond infection status. Clinically, these phenotypes can help stratify preoperative management intensity. Patients in the anemia-dominated phenotype may benefit from targeted optimization programs, including iron supplementation and hemoglobin correction prior to surgery. Those characterized by prolonged surgical duration may require senior surgeon involvement and operative time minimization, whereas elderly short-procedure patients may profit most from enhanced perioperative monitoring and geriatric co-management. Hence, each cluster represents a distinct modifiable risk context that could guide individualized perioperative planning. In practical terms, these phenotypic distinctions could be incorporated into structured perioperative care pathways. For example, preoperative assessment clinics may flag anemia-dominated patients for mandatory hemoglobin optimization to reach safe thresholds (e.g., >13 g/dL) prior to surgery, supported by intravenous iron supplementation or erythropoietin protocols. In addition to antiseptic preoperative washing of elective cases, early decolonization and antiseptic whole-body washing could also be initiated for urgent admissions such as femoral neck fracture patients upon arrival in the emergency department to reduce microbial load before arthroplasty. Restrictive use and early removal of urinary catheters should be emphasized, particularly in elderly short-procedure patients, to minimize infection risk and perioperative morbidity. In high-risk constellations, especially anemia-dominated or frail elderly phenotypes, training procedures should be strictly avoided, and the operation should be performed by the most experienced available surgeon to minimize intraoperative time and variability. Furthermore, a structured postoperative co-management involving both orthopedic surgeons and geriatricians should be established to ensure early mobilization, hemodynamic stability, and consistent infection monitoring. The early involvement of an antibiotic stewardship team is essential to guide prophylactic and therapeutic antibiotic use, reduce unnecessary exposure, and maintain microbiological vigilance in these vulnerable patient groups. Integrating phenotype-guided alerts into perioperative planning software could systematically align preventive action, including preoperative optimization, operative team selection, and postoperative follow-up, with the patient’s individual risk archetype.

The optimal cut-off analysis identified 49.6% probability as the balanced threshold (sensitivity 73.6%, specificity 80.2%). Despite only moderate discrimination (AUC 0.718; optimism-corrected AUC 0.661), the preoperative model demonstrated stable calibration and limited, yet consistent, net clinical benefit at low-risk thresholds in prevalence-weighted decision-curve analysis. This pattern suggests realistic model performance under typical preoperative data conditions and highlights feasibility over maximal predictive accuracy, which aligns with the clinical intent of early risk stratification. While the AUC values are modest, this was an intentional trade-off to preserve transparency and clinical interpretability. The model was optimized for clarity and real-world feasibility rather than maximal statistical accuracy, ensuring that each included variable has an understandable and actionable clinical meaning. We acknowledge that Youden’s index assumes equal misclassification costs and does not account for disease prevalence [5]. Therefore, performance metrics were evaluated across multiple thresholds to facilitate clinical interpretation under different risk tolerance scenarios. Within the matched cohort, our stratification identified three groups: low-risk patients, intermediate-risk patients, and high-risk patients. This stratification could provide a framework for developing further risk-adapted care pathways. However, clinical implementation requires model recalibration to population-level infection prevalence [7].

Our approach achieves clinically meaningful stratification using a model with five variables (age, anemia, CRP, ASA score, and surgical delay), substantially fewer than high-dimensional machine learning models in the literature, which feature 60+ variables [9,10]. The integration of clustering analysis with predictive modeling provides complementary perspectives; the regression models offer individualized probability estimates for patient-level decision-making, while phenotype-based clustering enables population-level stratification for system-wide resource planning and targeted care pathway development. Mathematical transparency through logistic regression ensures clinical interpretability and practical implementation feasibility. The case–control design provides valid estimates of relative risk associations (odds ratios) and enables effective risk stratification. The identified patient phenotypes provide a foundation for developing targeted intervention strategies that require prospective validation. Absolute risk prediction remains limited, requiring recalibration on a real-world population scale [7].Therefore, risk-reducing considerations for improved pre- and perioperative pathways can be outlined as follows.

For anemia-dominated cases, future studies should evaluate whether intensive preoperative optimization—including systematic hemoglobin correction, comorbidity stabilization, and consideration of staged procedures—reduces adverse outcomes, given the substantial intraoperative blood loss in these patients [20]. Low preoperative hemoglobin levels have repeatedly been associated with unfavorable outcomes in arthroplasty. Grammatopoulos et al. reported a nonlinear association between preoperative hemoglobin and postoperative morbidity, with optimal values around 135–140 g/L and a progressively increasing risk of transfusion and complications below 135 g/L [21]. Future investigations may confirm this range as a clinically relevant threshold for anemia-risk patients undergoing arthroplasty.

Furthermore, if high surgical duration is anticipated, patients may benefit from senior surgeon involvement and operative-time-minimization strategies. Elderly short-procedure patients may require enhanced perioperative monitoring and geriatric-focused care pathways to address age-related mortality risk. Implementation of fast-track protocols to minimize surgical delay represents a universal optimization target, given its association with prolonged hospitalization across all patient types. Risk-adapted prophylaxis strategies, including preoperative decolonization, statin administration, and preadmission skin preparation [22,23], warrant evaluation in randomized trials stratified by the identified risk categories. We acknowledge that our institutional definition of PJI, which is broader and more sensitive than the ICM/MSIS 2018 criteria, may have influenced the observed model performance. This approach likely increased case detection but also introduced some heterogeneity, potentially contributing to moderate AUC values. Nevertheless, the chosen definition reflects real-world diagnostic practice and preserves generalizability to clinical settings where comprehensive minor-criteria testing is not routinely available. To mitigate potential methodological bias arising from this broader definition, several statistical validation strategies were applied. Model calibration was assessed using the Hosmer–Lemeshow test and Brier score, and robustness was evaluated through 1,000-fold bootstrap internal validation to correct for optimism. In addition, decision curve analysis weighted to real-world infection prevalence (2%) was used to confirm the model’s clinical utility across different risk thresholds. These complementary approaches strengthen the reliability of our findings despite the inherent limitations of retrospective data and broadened diagnostic inclusion criteria.

Limitations can be identified as follows. This was a single-center study conducted at a tertiary academic institution, which inherently limits generalizability. Patient characteristics, perioperative protocols, and infection surveillance practices may differ across hospitals, so external validation in multicenter datasets is essential to confirm the stability and transferability of our findings. The 1:1 matched case–control design provides valid estimates of relative risk associations but limits direct prediction of absolute infection probabilities, which require recalibration to population-level prevalence before clinical implementation. The small number of mortality events (n = 10) reduces the model's statistical power for mortality prediction analysis. Mortality was analyzed as perioperative death, rather than time-to-event, which may not fully capture longer-term survival differences. This should be addressed in future and prospectively designed studies employing survival analysis methods with adequate follow-up duration. The single-center retrospective approach limits external validity. Thus, a multicenter validation is required before clinical implementation to confirm model performance and phenotype stability across different patient populations. While cohorts were successfully balanced for age and sex through 1:1 matching, resulting in a perfectly balanced case–control cohort of 182 patients (91 cases, 91 controls), we excluded 6 cases and 22 controls. This has the potential for introducing a selection bias. The clustering approach revealed distinct phenotypes in our cohort, but this requires validation in independent datasets to confirm reproducibility. The simplified preoperative model (excluding operative duration and postoperative antibiotics) achieved moderate discrimination (AUC = 0.718), demonstrating feasibility for preoperative risk stratification. Although both the preoperative (AUC = 0.718) and full model (AUC = 0.813) showed only moderate discrimination, these values represent realistic performance levels achievable in clinical datasets with limited preoperative information. The focus of this work was on transparency, interpretability, and clinical feasibility rather than maximizing predictive accuracy, aligning with the practical requirements of perioperative risk assessment. While prospective validation is still required, the model emphasizes real-world applicability and ease of clinical translation over maximal statistical performance. Though it requires separate prospective validation, the model prioritizes clinical feasibility and preoperative applicability over maximal predictive accuracy. A more complex model including intraoperative variables (such as operative duration) might achieve higher discrimination but would not be useful for preoperative risk stratification and optimization.

Model recalibration to population-level infection prevalence is required before the regression equation can generate clinically meaningful absolute risk predictions for individual patients. Prospective randomized controlled trials should evaluate whether phenotype-guided care pathways and risk-adapted interventions effectively reduce infection rates and improve outcomes compared to standard care. These trials should test specific hypotheses generated by our findings, such as whether intensive preoperative anemia correction reduces infection risk in anemia-dominated patients or whether enhanced geriatric care protocols reduce mortality in elderly short-procedure patients. The implementation of the model as a digital clinical decision support tool integrated into electronic health record systems would facilitate widespread adoption and enable real-world outcome monitoring through learning health system approaches. Such tools should be prospectively evaluated before implementation as standard practice. Longitudinal studies can examine the impact of targeted interventions on phenotype migration and whether patients can be successfully transferred from higher-risk to lower-risk phenotypes. Collectively, these clusters represent clinically recognizable patient archetypes that may be used to tailor perioperative strategies. Translating these phenotypes into preoperative risk categories could help to allocate preventive resources more efficiently and personalize perioperative decision-making.

## 5. Conclusions

This study identifies preoperative anemia, ASA score, and surgical delay as critical independent preoperative risk factors while revealing three distinct patient phenotypes with divergent clinical trajectories. The integration of predictive modeling with clustering analysis provides both individualized risk estimation and population-based stratification, achieved with a five-parameter model. These findings establish a proof-of-concept framework for risk-stratified perioperative care. However, clinical implementation requires external validation, and model recalibration to real-world prevalence is needed. Furthermore, a prospective evaluation through randomized controlled trials should be conducted to determine whether phenotype-guided pathways for systematic preoperative optimization and risk-adapted prophylaxis strategies can reduce infection rates and improve patient outcomes. The current study provides the foundational evidence and analytical framework necessary for these next-stage validation studies. Given the single-center design, external validity and generalizability to other institutions and patient populations remain limited. Future multicenter studies are warranted to validate these findings across different clinical settings.

## Figures and Tables

**Table 3:** Patient phenotype characteristics and clinical outcomes. Comprehensive comparison of demographic, surgical, and outcome variables across the three identified patient phenotypes. Values are presented as means ± standard deviations for continuous variables and percentages for categorical variables. All between-group differences were statistically significant (*p* < 0.001).

Cluster	N	Percentage	Age (Years)	Surgery Duration (min)	Anemia (%)	CRP (mg/L)	ASA Score	Perioperative Mortality (%)
High Surgery Duration	47	25.8%	62.7 ± 9.5	159.0 ± 94.9	2.1%	3.9 [2.0–8.0]	2	0.0%
Elderly Short-Procedure	112	61.5%	75.9 ± 10.1	109.8 ± 54.5	0.0%	4.2 [2.2–12.4]	3	6.2%
Anemia-Dominated	23	12.6%	71.9 ± 9.9	175.6 ± 84.2	95.7%	46.8 [16.6–59.0]	3	13.0%

**Table 4:** Independent risk factors for periprosthetic joint infection: multivariable logistic regression analysis. Results of multivariable logistic regression identifying significant preoperative predictors of periprosthetic joint infection. The model includes only variables available before surgery to enable preoperative risk stratification and optimization. Operative duration (unknown preoperatively) and postoperative antibiotic prophylaxis (confounded by indication) were intentionally excluded. Odds ratios (OR) are presented with 95% confidence intervals (CI) and *p*-values.

Variable	Odds Ratio	95% CI	*p*-Value
Age (log)	0.42	0.06–3.05	0.3895
Anemia	5.91	1.23–28.37	0.0263
CRP (log)	1.13	0.89–1.45	0.3190
ASA score	1.77	1.05–2.99	0.0331
Delay (log)	1.67	1.07–2.62	0.0238

**Table 5:** Predictive performance metrics at different probability thresholds. Sensitivity and specificity across different cut-off thresholds for infection probability. The analysis demonstrates the trade-off between sensitivity and specificity, with 50% threshold providing optimal balance according to Youden’s index.

Threshold	Sensitivity	Specificity	True Positives	True Negatives	False Positives	False Negatives
10%	96.7%	4.4%	88	4	87	3
25%	87.9%	38.5%	80	35	56	11
50%	72.5%	80.2%	66	73	18	25
75%	44.0%	96.7%	40	88	3	51

## Data Availability

The datasets generated and analyzed during the current study are not publicly available due to patient privacy and data protection regulations (GDPR/EU data protection law) but are available from the corresponding author on reasonable request and subject to appropriate data sharing agreements and ethics approval.
